# Serum levels of B-cell activating factor are associated with a reduced risk of chronic lymphocytic leukemia

**DOI:** 10.1038/s41408-024-01106-7

**Published:** 2024-08-07

**Authors:** Eleanor Frost, Jonathan N. Hofmann, Wen-Yi Huang, Ashley A. Frazer-Abel, Kevin D. Deane, Sonja I. Berndt

**Affiliations:** 1 Department of Epidemiology & Population Health, Stanford University School of Medicine, Stanford, CA USA; 2 Division of Cancer Epidemiology and Genetics, National Cancer Institute, Bethesda, MD USA; 3 Department of Medicine, University of Colorado Anschutz Medical Campus, Denver, CO USA

**Keywords:** Cancer epidemiology, Chronic lymphocytic leukaemia

## Abstract

- Immune dysregulation is thought to contribute to chronic lymphocytic leukemia (CLL) risk, but biological mechanisms are unclear. We discovered that increased serum levels of B-cell activating factor (BAFF), an important regulator of B-cell maturation, were associated with a decreased risk of CLL, even >10 years after blood draw. Our findings suggest that BAFF could be a useful biomarker to assess risk among individuals at high risk, such as those with monoclonal b-cell lymphocytosis.

Immune dysregulation and altered immune function have been implicated in the etiology of chronic lymphocytic leukemia (CLL). Autoimmune diseases, such as rheumatoid arthritis, have been associated with an increased risk of lymphoid malignancies [1, 2], potentially through activation of the B-cell receptor signaling pathway, and autoimmune conditions have been reported as complications of CLL [3]. Atopic diseases and related conditions have been associated with a reduced risk of CLL in retrospective studies [4]. Prospective studies have observed lower plasma levels of IgE to be associated with increased risk of CLL [5, 6], implicating impaired immune function in CLL risk; however, the biologic mechanisms by which these immune-related conditions alter risk are unclear.

We sought to clarify the association between immune dysregulation and CLL risk and explore potential underlying mechanisms by examining pre-diagnostic serum immune biomarkers and CLL risk in a prospective nested case-control study. We focused on B-cell activating factor (BAFF), a member of the TNF family that regulates B-cell differentiation and maturation and has been linked to autoimmunity [7] and atopic diseases [8, 9], and biomarkers commonly measured in the diagnostic evaluation of rheumatoid arthritis: anti-cyclic citrullinated peptide-3 antibody (anti-CCP3) and two rheumatoid factors (RF-IgM and RF-IgA).

For this nested case-control study, we utilized participants from the intervention arm of the Prostate, Lung, Colorectal and Ovarian (PLCO) Cancer Screening Trial, a randomized clinical trial including over 154,000 participants aged 55–74 years. Subjects randomized to the intervention arm (*N* = 77,444) completed a baseline risk factor questionnaire and provided blood specimens at baseline (T0) and subsequent screening exams. Participants were actively followed for cancer incidence and mortality through 2011, and medical records were abstracted to confirm cancer diagnoses. Subjects provided written informed consent, and the study was approved by the institutional review board. As described previously [10], PLCO participants were eligible for this case-control study if they completed a baseline questionnaire, had stored pre-diagnostic serum available, and consented to participate in etiologic studies. Individuals with a prior history of cancer were excluded. A total of 832 participants developed a lymphoid malignancy, including 221 with CLL or small lymphocytic lymphoma. Controls (*n* = 809) were required to be cancer-free and alive at the time of case diagnosis and were matched to cases with lymphoid malignancies on age (+/− 1 year), race, gender, calendar year of blood draw (e.g., 1993), and study year of blood draw (e.g., T0).

Using pre-diagnostic stored serum, BAFF serum levels were measured using BAFF/BLyS/TNFSF13B Quantikine ELISA kit (R&D Systems), and anti-CCP3, RF-IgM, and RF-IgA levels were measured with QUANTA Lite assays (Inova Diagnostics). Assays demonstrated high reproducibility with coefficients of variation <7%.

Logistic regression was used to determine the odds ratios (ORs) and 95% confidence intervals (95% CIs) for the association between the analytes and CLL risk. The biomarkers were analyzed as quantitative traits and using clinical laboratory cut-points and/or quintiles based on the distribution among controls. Anti-CCP3 was rank-based inverse normal transformed and BAFF was log_2_ transformed to achieve normal distributions. Matching factors (age, gender, race, calendar and study year of the blood draw), number of freeze-thaw cycles, and batch were included in the logistic models. Other potential confounders, such as smoking, did not alter the associations observed and were not included in the final models. To determine whether demographic or lifestyle factors modified the relationship with CLL risk, we conducted stratified analyses and tested for interaction using a Wald test. Sensitivity analyses were performed excluding those with self-reported autoimmune disease (i.e., rheumatoid arthritis, Crohn’s disease, or ulcerative colitis) (*n* = 63).

CLL cases and controls were similar across most baseline risk factors, although cases were more likely to be non-Hispanic white (Supplementary Table 1). The median time from blood draw to diagnosis for cases was 6.4 years [interquartile range (IQR): 3.7–8.8] with a median age at diagnosis of 70 years (IQR: 65–75). Serum levels of BAFF and anti-CCP3 were lower for cases compared to controls (BAFF: 562.0 pg/ml vs. 640.2 pg/ml, *P* = 0.003; anti-CCP3: 9.66 u/ml vs. 10.68 u/ml, *P* = 0.001, for cases and controls, respectively). Similar proportions of cases and controls were high positive for RF-IgM (10.9% vs. 11.5%, respectively) and RF-IgA (4.5% vs. 4.5%, respectively).

Multivariate regression analyses demonstrated that higher BAFF serum levels were associated with a significant reduction in CLL risk (*P*_*trend*_ = 5 × 10^−6^, Fig. 1a), which appeared linear (Supplementary Fig. 1). The highest quintile of BAFF was associated with an 80% reduction in CLL risk compared to the lowest quintile (*OR*_*5th* vs. 1st quintile_ = 0.20, 95% CI: 0.11–0.37, *P* = 1.0 × 10^−4^). The results were similar after excluding individuals with autoimmune disease, adjusting for anti-CCP3, RF-IgM, and RF-IgA, or limiting the dataset to individually matched cases and controls (Supplementary Tables 2 and 3). Stratified analyses showed no evidence of effect modification by age, sex or other factors (Fig. 2a, *P*_*interaction*_ > 0.05 for all). The association with BAFF was similar regardless of the number of years from blood draw to diagnosis for the cases (Fig. 2a), suggesting that early CLL disease was unlikely to explain the reduced BAFF levels observed for cases. Even after restricting the analysis to cases diagnosed > 10 years after blood draw, BAFF remained protective for CLL (*OR*_*continuous*_ = 0.40, 95% CI: 0.19–0.83, *P* = 0.01).

**Fig. 1:**
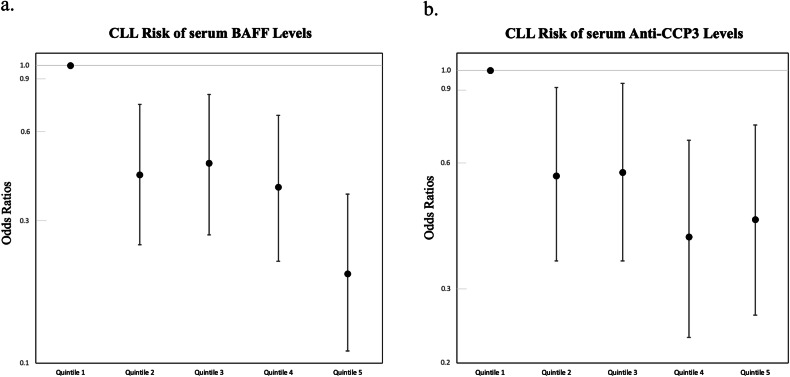
Risk of CLL associated with quintiles of serum analytes. BAFF quintiles are shown in (**a**) and anti-CCP3 quintiles are shown in (**b**). Quintiles for BAFF and anti-CCP3 were created based on levels observed among controls. Anti-CCP3 quintile ranges were: <8.31, >8.31 and <9.84, >9.84 and <11.95, >11.95 and <17.0, and >17.0 u/ml. BAFF quintile ranges were: <435, >435 and <556, >556 and <706, >706 and <890, and >890 pg/ml. Odds ratios and 95% CIs were estimated using logistic regression and adjusting for age, gender, race, year of blood draw, number of freeze thaw cycles, and batch.

**Fig. 2:**
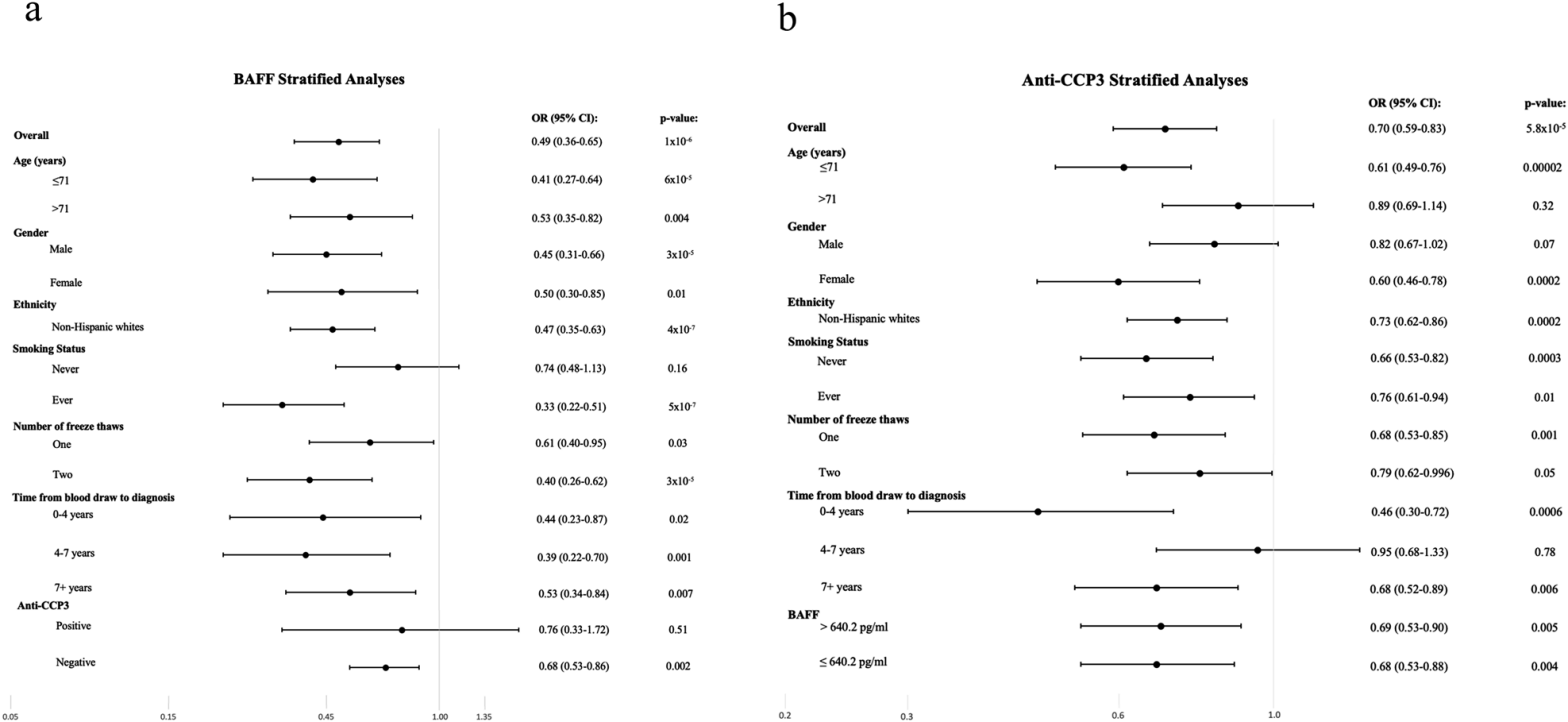
Risk of CLL associated with serum levels of analytes stratified by select characteristics. BAFF stratified analyses are shown in (**a**) and anti-CCP3 stratified analyses are shown in (**b**). Reported ORs (95% CI) for association of BAFF and anti-CCP3 in relation to CLL risk were estimated using logistic regression and adjusting for age, gender, race, year of blood draw, number of freeze thaw cycles, and batch. Anti-CCP3 was stratified by the median BAFF of controls (640.2 pg/ml). There were not enough individuals to estimate the associations for non-Hispanic whites separately. None of the differences in ORs between levels within each of the stratified factors were statistically significant (*P*_interaction_ > 0.05).

Elevated levels of anti-CCP3 were also associated with a reduced risk of CLL (*P*_*trend*_ = 5 × 10^−4^, Fig. 1b) with the highest quintile of anti-CCP3 showing a 56% reduced CLL risk compared to the lowest quintile (OR_5th vs. 1st quintile_ = 0.44, 95% CI: 0.26–0.74, *P* = 0.002). When clinical cutpoints were used for analysis, those testing positive for anti-CCP3 (≥20 Units) had a reduced risk of CLL (OR 0.45, 95% CI: 0.24–0.81, *P* = 0.009). The results were similar when individuals with autoimmune disease were excluded, after adjustment for BAFF, RF-IgA, and RF-IgM, and when the analysis was restricted to the matched cases and controls (Supplementary Tables 2 and 3). No significant differences in risk were observed by age, sex, or other factors (Fig. 2b, P_interaction_ > 0.05 for all). When the analysis was restricted to cases diagnosed > 10 years after blood draw, anti-CCP3 appeared to remain protective for CLL but was not statistically significant (OR_continuous_ = 0.73, 95% CI: 0.46–1.16, *P* = 0.18). RF-IgM and RF-IgA were not significantly associated with risk of CLL (Supplementary Tables 2 and 3).

In this prospective, nested case-control study, increased serum levels of BAFF were associated with a reduced risk of developing CLL that persisted for >10 years after blood draw. Our findings are consistent with previous studies [11–16], including a prospective study that also observed a protective association more than a decade after blood draw [11], providing further evidence that BAFF may be an etiologic risk factor of CLL.

BAFF plays a key role in promoting B-cell differentiation, maturation, and survival. Although one might have expected higher BAFF levels to be associated with an increased risk of CLL because BAFF promotes the survival of CLL clones through the canonical NF-κB pathway, this pathway is not used in normal B-cells [17]. Instead BAFF promotes the differentiation of immature transitional cells into mature B-cells [18]. Mice that lack BAFF show normal B-cell development up to the transitional phase, but further maturation beyond the T1 B-cell stage is hindered [19]. In humans, higher BAFF levels have been associated with lower immature/naive CD5 + B-cells in infants and children and higher proportions of mature CD27+ memory B-cells [20]. By promoting maturation, increased BAFF levels may reduce the availability of early-stage B-cells, decreasing the likelihood that one of those immature cells acquires pathologic alterations leading to a malignant CLL clone.

Interestingly, elevated levels of BAFF have been linked to smoking, dairy farming, and atopic conditions [8, 9, 20], all of which have been shown to be associated with a reduced risk of CLL [4], raising the possibility that BAFF levels may be a contributing factor. We observed elevated BAFF levels among current smokers in our study, but the association between serum BAFF levels and risk of CLL remained the same after adjustment for smoking, and no differences were observed when we stratified by smoking status. Information on atopic conditions was not available in our study.

We also observed a reduced risk of CLL with higher anti-CCP3 levels; however, no association was observed with the rheumatoid factors. Higher anti-CCP3 may reflect a specific type of altered immune function. Rheumatoid arthritis patients who are positive for anti-citrullinated protein autoantibodies have altered immune and B-cell subpopulation profiles with lower proportions of naïve B-cells compared to patients who are negative [21].

Our study population was limited predominantly to older non-Hispanic whites, and therefore, our results may not be generalizable to other populations. Participants in our study may have had monoclonal b-cell lymphocytosis (MBL) at the time of the blood draw, potentially contributing to the inverse association observed. However, if MBL was responsible for the inverse association with BAFF, one would have expected the association to become stronger closer to diagnosis as clones expanded and we did not observe evidence for this. Our study is the largest prospective study conducted to date of serum BAFF levels and CLL risk. The collection of blood samples years prior to CLL diagnosis limited the potential for reverse causality and selection bias, making an important contribution to our understanding of risk factors for CLL.

In conclusion, increased serum levels of BAFF and anti-CCP3 were associated with a decreased risk of CLL. Although the results of this study need to be replicated, our study demonstrates that BAFF, an important regulator of B-cell maturation, may be important in the etiology of CLL. Future studies should investigate whether serum BAFF levels are predictive of CLL development among individuals at high risk of CLL, such as those with a family history of CLL or a diagnosis of MBL.

### Supplementary information

Supplementary Table 1

Supplementary Table 2

Supplementary Table 3

Supplementary Figure 1

## Data Availability

Data used in this study are available for research purposes upon request and approval from the PLCO Cancer Data Access System (https://cdas.cancer.gov/plco/).
