# Supplementary material for: Serum levels of B-cell activating factor are associated with a reduced risk of chronic lymphocytic leukemia
Source: Blood Cancer J. 2024 Aug 7;14(1):132. doi: 10.1038/s41408-024-01106-7 (PMC11306784; doi:10.1038/s41408-024-01106-7)
Supplement: Supplementary file 4 — Supplementary Figure 1 [file 41408_2024_1106_MOESM4_ESM.docx]

**Supplementary Figure 1**. Restricted cubic spline model for the association between log_2_-transformed BAFF and CLL risk. Log_2_-transformed BAFF was modeled as a restricted cubic spline with 3 knots placed using Harrell’s recommended percentiles. The restricted cubic spline model was not found to fit the data better than the linear model (P=0.93).
